# Long-Term Outcomes of a Kahook Dual Blade Procedure Combined with Phacoemulsification in Japanese Patients with Open-Angle Glaucoma

**DOI:** 10.3390/jcm11051354

**Published:** 2022-03-01

**Authors:** Kentaro Iwasaki, Hiroshi Kakimoto, Yusuke Orii, Shogo Arimura, Yoshihiro Takamura, Masaru Inatani

**Affiliations:** 1 Department of Ophthalmology, Faculty of Medical Sciences, University of Fukui, Fukui 910-1193, Japan; kenkentaro0329@yahoo.co.jp (K.I.); orii@u-fukui.ac.jp (Y.O.); leosunshine33@gmail.com (S.A.); ytakamura@hotmail.com (Y.T.); 2 Department of Ophthalmology, Obama Hospital, Fukui 917-0078, Japan; hrs0214k@gmail.com

**Keywords:** Kahook dual blade, phacoemulsification, open-angle glaucoma, surgical efficacy, surgical complication

## Abstract

We retrospectively evaluated the long-term surgical outcomes of phacoemulsification combined with a Kahook dual blade (KDB) procedure in Japanese patients with open-angle glaucoma. The primary outcome was surgical success or failure. Surgical failure was indicated by a <20% reduction in preoperative intraocular pressure (IOP) or IOP > 18 mmHg (criterion A), IOP > 14 mmHg (criterion B), or requirement for reoperation. Glaucoma medications after surgery and postoperative complications were recorded. Surgical outcomes were compared between primary open-angle glaucoma (POAG) and exfoliation glaucoma (ExG) groups. The probability of success at 36 months postoperation was 52.5% using criterion A and 36.9% using criterion B. Mean IOP decreased significantly from 19.5 ± 6.9 mmHg preoperatively to 11.9 ± 2.7 mmHg at 36 months, and the mean number of glaucoma medications from 2.4 ± 1.4 to 1.6 ± 1.4 (both *p* < 0.01). IOP spikes were significantly more common in the ExG group (23.7% vs. 9.1%; *p* = 0.045), as was the need for additional glaucoma surgery (10.5% vs. 1.8%; *p* = 0.038). A KDB procedure combined with cataract surgery resulted in significant long-term decreases in IOP and the number of glaucoma medications. The complication rate was higher in eyes with ExG. Therefore, these eyes require more careful management after a KDB procedure.

## 1. Introduction

Trabeculotomy is effective for the reduction in intraocular pressure (IOP) in patients with glaucoma [1,2,3,4]. The trabecular meshwork and inner walls of Schlemm’s canal are the main sites of resistance to aqueous outflow [1,5,6]. During trabeculotomy, removal of that resistance results in reduction in IOP. Conventional trabeculotomy can be performed using an ab externo approach with metal trabecular probes, which requires conjunctival and scleral incisions and sutures. This approach is invasive and leads to obstruction whenever additional trabeculectomy is needed [7,8,9]. Newer techniques performed using an ab interno approach are becoming popular in Japan due to their minimal invasiveness [10]. Compared with the ab externo approach, ab interno trabeculotomy surgery is safe and has a short recovery time [11,12,13].

The Kahook dual blade (KDB; New World Medical, Rancho Cucamonga, CA, USA) is suitable for use in ab interno trabeculotomy surgery and is designed to excise a strip of the trabecular meshwork and the inner walls of Schlemm’s canal using dual blades to decrease resistance to aqueous outflow [14]. Ab interno trabeculotomy surgery is often performed in combination with cataract surgery, and we have previously reported the 12-month surgical outcome of the KDB procedure combined with cataract surgery [15]. Although many studies on the surgical outcomes of the KDB procedure have been published [16,17,18,19,20,21,22], few have reported the long-term outcomes when this procedure is combined with cataract surgery [23]. One report suggests that conventional trabeculotomy is more effective in eyes with exfoliation glaucoma (ExG) than in those with primary open-angle glaucoma (POAG) [1]. Another study suggests that a stand-alone KDB procedure is more effective for eyes with ExG than for those with POAG [24]. A third report suggests that a KDB procedure achieves greater IOP reduction in eyes with ExG than in eyes with POAG, irrespective of whether it is combined with cataract surgery [25]. However, all these studies had small sample sizes. The surgical outcomes of combined KDB and cataract surgery have not been compared between POAG and ExG. Therefore, the aim of the current study is to evaluate the long-term surgical outcomes of phacoemulsification combined with a KDB procedure in Japanese patients and to compare the surgical outcomes of this combination between eyes with POAG and those with ExG.

## 2. Materials and Methods

### 2.1. Patient Selection

This retrospective clinical cohort study analyzed data from patients treated with phacoemulsification in combination with a KDB procedure at the University of Fukui Hospital or Obama Hospital in Japan between February 2017 and October 2018. The study’s inclusion criteria were those older than 19 years, presence of either POAG or ExG, and no history of intraocular surgery. Data for patients with primary angle closure glaucoma, neovascular glaucoma, secondary glaucoma (except ExG), and congenital glaucoma were excluded. Data for 148 eyes of 97 patients who underwent combined phacoemulsification with KDB surgery were available for analysis.

### 2.2. Surgical Technique

All surgeries were performed by experienced glaucoma specialists, and the surgical procedures were as previously described [15]. Standard phacoemulsification was performed with implantation of an intraocular lens through a clear 2.4-mm temporal corneal incision using topical anesthesia. The patient’s head and the microscope were tilted to visualize the nasal angle with a gonioprism. The surgeon then filled the anterior chamber with additional viscoelastic material (1% sodium hyaluronate, Opegan Hi, Santen Pharmaceutical, Osaka, Japan). Next, the tip of the KDB was inserted into the Schlemm’s canal and moved circumferentially to excise the trabecular meshwork over 3–4 clock hours. Finally, the viscoelastic material was removed, and the anterior chamber was filled with a balanced saline solution.

### 2.3. Postoperative Care and Data Collection

All patients received similar postoperative topical medications, namely, 0.3% gatifloxacin 3 times per day for 1–2 weeks and 0.1% betamethasone phosphate 3 times per day for 3–4 weeks. Glaucoma medications were stopped at the time of the surgery and resumed according to the surgeon’s discretion at postoperative follow-up visits. We collected data on patient demographics and clinical characteristics, including sex, age, type of glaucoma, best corrected visual acuity (BCVA), spherical equivalent, axial length, severity of glaucoma, preoperative IOP, postoperative IOP, the number of glaucoma medications administered, and any postoperative complications. A logarithm of the reciprocal of the decimal BCVA was used to approximate the logarithm of the minimal angle of resolution (logMAR). Eyes without form vision were classified into 1 of 4 low-vision categories, which were assigned decimal equivalents as follows: counting fingers 0.00500, hand motions 0.00250, light perception 0.00125, and no light perception 0.00010 [26]. We classified the severity of glaucoma into three stages using the mean defect (MD) score on Humphrey visual field tests (MD ≥ −6.0 dB, mild; −6.0 > MD ≥ −12.0 dB, moderate; and MD < −12.0 dB, severe). In terms of postoperative complications, we defined hyphema as formation of a blood niveau in the anterior chamber, and an IOP spike as an increase in IOP of >10 mmHg above baseline within 1 month after surgery.

### 2.4. Outcome Measures

The primary outcome was surgical success or failure, which was defined according to two IOP criteria. Failure was defined according to the postoperative IOP level with or without glaucoma medication at ≥1 month after surgery as follows: <20% reduction in the preoperative IOP value or an IOP > 18 mmHg (criterion A) and an IOP > 14 mmHg (criterion B) on two consecutive follow-up visits. We also deemed cases that required reoperation for glaucoma as surgical failures. All cases that did not meet the criteria for failure were deemed to be successful. The secondary outcomes included IOP, the number of glaucoma medications used, postoperative complications, visual acuity, and prognostic factors for surgical failure.

### 2.5. Statistical Analysis

Univariable comparisons between groups were performed using the chi-square test, Fisher’s exact test, paired t-test, and unpaired t-test with Bonferroni correction. The probability of success was analyzed using Kaplan–Meier survival curves and compared between groups using the log-rank test. Multivariable analysis was performed to determine the prognostic factors for failure of surgery using Cox proportional hazards models. Reductions in IOP and medication scores were also analyzed using the linear mixed model, wherein the patients were regarded as the random effect and time period as a fixed effect to reduce the possible bias of including both eyes in the same patient. *P*-values < 0.05 were considered statistically significant. If the difference in surgical success rate between the POAG and ExG group was ≥35% [24] with a two-sided significance level of 0.05 and a power of 0.8. The estimated sample size of at least 36 eyes in each group was considered as essential to detect a significant difference between the two groups.

## 3. Results

### 3.1. Patient Demographic and Clinical Characteristics

Data were analyzed for 148 eyes of 97 patients who underwent phacoemulsification combined with KDB surgery. The mean follow-up duration was 31.3 ± 14.8 months for all eyes. The 148 eyes were classified into the POAG group (n = 110) and the ExG group (n = 38). Table 1 summarizes the patients’ baseline characteristics. There was no significant between-group difference in the preoperative status. The mean follow-up duration was 31.8 ± 14.4 months in the POAG group and 30.1 ± 16.1 months in the ExG group (*p* = 0.70).

### 3.2. Primary Outcome

**Figure 1:**
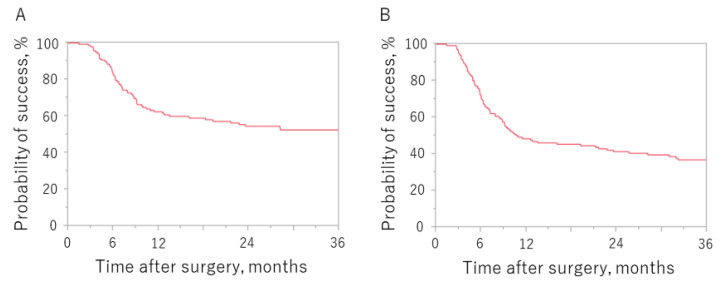
Kaplan–Meier survival curves according to which failure criterion was used. Criterion (**A**): IOP > 18 mmHg, <20% reduction in preoperative IOP value, or reoperation for glaucoma. Criterion (**B**): IOP > 14 mmHg, <20% reduction in preoperative IOP value, or reoperation for glaucoma. IOP, intraocular pressure.

Figure 1 shows the Kaplan–Meier survival curves for surgical outcomes according to whether criterion A or B was used to define failure. The probability of success at 12, 24, and 36 months postoperation was 62.4%, 54.6% and 52.5%, respectively, for criterion A and 48.5%, 41.4% and 36.9%, respectively, for criterion B.

**Figure 2:**
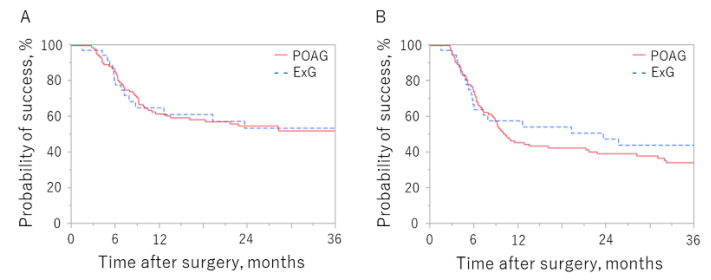
Kaplan–Meier survival curves in the POAG and ExG groups according to which criterion was used to define failure. Criterion (**A**): IOP > 18 mmHg, <20% reduction in preoperative IOP value, or reoperation for glaucoma. Criterion (**B**): IOP > 14 mmHg, <20% reduction in preoperative IOP value, or reoperation for glaucoma. ExG, exfoliation glaucoma; IOP, intraocular pressure; POAG, primary open-angle glaucoma.

Figure 2 shows the Kaplan–Meier survival curves comparing surgical outcomes in the POAG and ExG groups according to whether criterion A or B was used. There was no significant between-group difference using either criterion. The probability of success at 36 months in the POAG and ExG groups was 52.2% and 53.7%, respectively, for criterion A (*p* = 0.99) and 34.4% and 44.2%, respectively, for criterion B (*p* = 0.50).

The cumulative success rate was 62.4%, 54.6%, and 52.5% at 12, 24, and 36 months postoperation, respectively, using criterion A, and 48.5%, 41.4% and 36.9%, respectively, using criterion B.

The cumulative success rates in the POAG and ExG groups at 36 months after surgery were 52.2% and 53.7%, respectively, using criterion A (*p* = 0.99) and 34.4% and 44.2%, respectively, using criterion B (*p* = 0.50).

### 3.3. Secondary Outcomes

**Figure 3:**
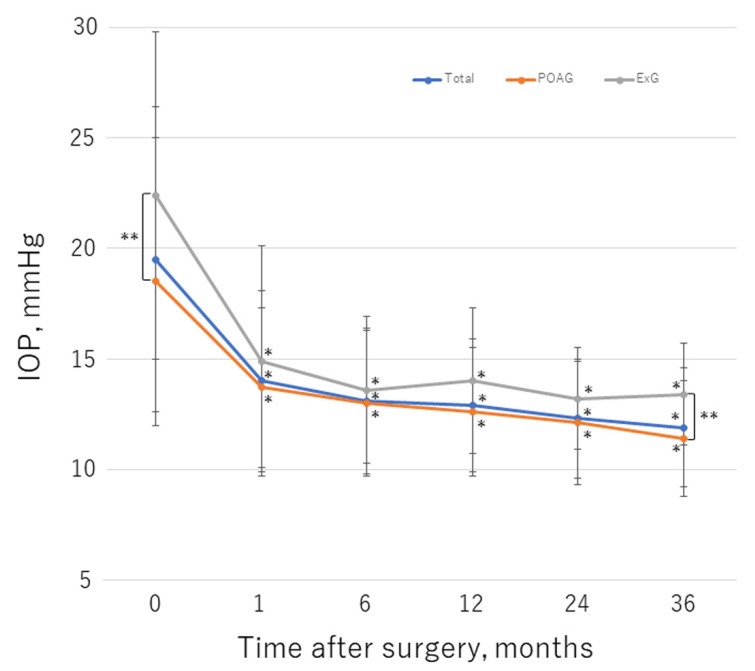
Changes in mean IOP over the course of follow-up. *, statistically significant difference between the preoperative value and each time point. **, statistically significant difference between the POAG and ExG group. Error bar shows the standard deviation. ExG, exfoliation glaucoma; IOP, intraocular pressure; POAG, primary open-angle glaucoma.

**Figure 4:**
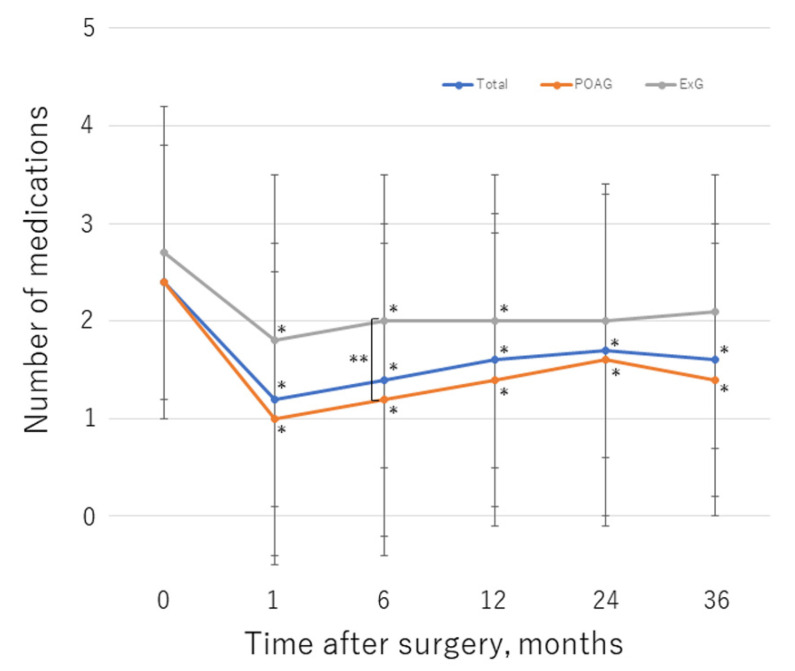
Changes in mean number of glaucoma medications over the course of follow-up. *, statistically significant difference between the preoperative value and each time point. **, statistically significant difference between the POAG and ExG group. Error bar shows the standard deviation. ExG, exfoliation glaucoma; POAG, primary open-angle glaucoma.

Table 2 shows the IOP values and numbers of glaucoma medications used at follow-up time points. With the linear mixed model, the postoperative changes in IOP and the number of glaucoma medications were significant in the entire dataset, and in eyes with POAG or ExG (*p* < 0.01 for each model). Figure 3 and Figure 4 illustrate the change in mean IOP and glaucoma medications, respectively, over the course of follow-up for each group. Before surgery, the mean IOP was 19.5 ± 6.9 mmHg and a mean number of 2.4 ± 1.4 glaucoma medications were used; these values decreased significantly to 11.9 ± 2.7 mmHg (*p* < 0.01) and 1.6 ± 1.4 (*p* < 0.01), respectively, by 36 months postoperation. In the POAG group, the mean preoperative IOP was 18.5 ± 6.5 mmHg, with a mean number of 2.4 ± 1.4 glaucoma medications used; these values decreased significantly to 11.4 ± 2.6 mmHg (*p* < 0.01) and 1.4 ± 1.4 (*p* < 0.01), respectively, by 36 months after surgery. In the ExG group, the mean preoperative IOP was 22.4 ± 7.4 mmHg with a mean number of 2.7 ± 1.5 glaucoma medications used; these values decreased to 13.4 ± 2.3 mmHg (*p* < 0.01) and 2.1 ± 1.4 (*p* = 0.38), respectively, by 36 months postoperation. There was no significant change in the number of medications used in the ExG group. The IOP was significantly higher in the ExG group than in the POAG group preoperatively (*p* < 0.01) and at 36 months postoperation (*p* = 0.014). At 6 months after surgery, significantly fewer glaucoma medications were used in the POAG group than in the ExG group (*p* = 0.039). IOP decreased by 28.0% in the study group overall, by 28.7% in the POAG group, and by 26.0% in the ExG group by 36 months postoperation; the between-group difference was not statistically significant (*p* = 0.47). At the final visit, an IOP reduction of ≥20% from baseline was achieved in 105 eyes (71%) overall, 81 eyes (74%) in the POAG group, and 24 eyes (63%) in the ExG group; the difference between the POAG and ExG groups was not significant (*p* = 0.22). The medication-free rate overall was 39%, 36%, and 34% at 12, 24, and 36 months postoperation, respectively. The medication-free rate was higher in the POAG group than in the ExG group; the between-group difference was significant at 12 months postoperation (*p* = 0.046). Compared with baseline, fewer medications were required at the final visit in 83 eyes overall (56%), 67 eyes (61%) in the POAG group, and 16 eyes (42%) in the ExG group; the between-group difference was not significant (*p* = 0.058).

Table 3 summarizes the postoperative complications. Hyphema occurred in 14.2% of cases overall but cleared spontaneously without intervention in all cases. IOP spikes were significantly more common in the ExG group than in the POAG group (23.7% vs. 9.1%; *p* = 0.045). Additional glaucoma surgery was required in significantly more cases in the ExG group (10.5%; trabeculectomy in three eyes, tube shunt surgery in one eye) than in the POAG group (1.8%; trabeculectomy in two eyes; *p* = 0.038). There were no complications related to cataract surgery.

The mean BCVA (LogMAR) was siginificantly improved at the final follow-up visit, from 0.53 ± 0.46 to 0.35 ± 0.67 (*p* < 0.01). In the POAG group, the mean BCVA was siginificantly improved at the final follow-up visit, from 0.56 ± 0.51 to 0.35 ± 0.65 (*p* < 0.01). In the ExG group, the mean BCVA was improved at the final follow-up visit, from 0.44 ± 0.27 to 0.36 ± 0.75, although the difference was not siginificant (*p* = 0.59).

The patients’ characteristics, including sex, age, axial length, visual field mean defect, glaucoma type, preoperative IOP, the number of preoperative glaucoma medications, and postoperative IOP spikes were evaluated as possible determinants of surgical failure. Table 4 shows the analyses using the multivariable Cox proportional hazards regression models. Lower preoperative IOP was associated with poorer surgical outcomes for criterion A (*p* < 0.01).

## 4. Discussion

This study evaluated the long-term surgical outcomes of combined KDB and cataract surgery and compared these outcomes between eyes with POAG and eyes with ExG. The probability of success at 36 months after surgery was 52.5% using criterion A and 36.9% using criterion B. The KDB procedure resulted in a significant reduction in IOP and the number of glaucoma medications required during 36 months of follow-up. There was no significant difference in the probability of success at 36 months after surgery between the POAG and ExG groups using either criterion. IOP spikes and additional glaucoma surgery were significantly more common in the ExG group than in the POAG group.

There are many reports on the surgical outcomes (IOP, the number of glaucoma medications used, complications) of a KDB procedure combined with cataract surgery at 6 [16,20], 12 [17,19,21,22], and 24 months [23]. However, these studies shared the limitation of small sample sizes. Our present study is unique in that it evaluated the long-term (36-month) surgical success rate using survival curves in a larger group of eyes with open-angle glaucoma (n = 148) and directly compared the surgical outcomes between eyes with POAG and eyes with ExG.

We evaluated the probability of success of combined KDB and cataract surgery through to 36 months postoperation. The probability of success was 52.5% for an IOP of <18 mmHg and 36.9% for an IOP of <14 mmHg. When conventional ab externo trabeculotomy was combined with cataract extraction, the probability of success was 29.5% for an IOP > 17 mmHg and 13.5% for an IOP > 15 mmHg, 3 years after surgery [6]. These long-term follow-up data indicate that a KDB procedure is more effective than conventional ab externo trabeculotomy. The incision of the trabecular meshwork strip made by a metal trabecular probe in conventional trabeculotomy might result in early adhesion after surgery, unlike the complete excision of the trabecular meshwork strip made possible using the KDB.

The probability of success after combined KDB and cataract surgery was similar between the POAG and ExG groups in our study. A previous study found no significant difference in the probability of success after KDB goniotomy with and without phacoemulsification between eyes with POAG and eyes with ExG [27]. Other studies showed that KDB surgery was more effective in eyes with ExG than in those with POAG [24,25]. The inconsistencies between the findings of our study and those of previous studies may stem from differences in sample size, patient background characteristics (especially severity of glaucoma), and the surgical procedures used (stand-alone, combined surgery, or stand-alone and combined surgery).

Previous studies suggested respective reductions in IOP and the number of glaucoma medications used by 12.7–32.1% and 21.1–62.7% after combined KDB and cataract surgery [16,17,23,25,27]. The decreases in IOP and the number of medications used which were observed in our study are consistent with those in previous studies. Although the IOP value was significantly higher in the ExG group than in the POAG group at 36 months postoperation, the rates of reduction in IOP from baseline were similar between the two groups. The patients in our ExG group required more glaucoma medications than those in our POAG group at all follow-up time points, which is consistent with a previous report [25]. This finding might be explained by the greater number of cases of severe glaucoma in the ExG group in which more medications may have been needed to maintain the IOP postoperatively. Another explanation may be the difference in the frequency of IOP spikes, which were more common in the ExG group than in the POAG group (23.7% vs. 9.1%). Due to this, the ExG group may have required more glaucoma medications to control IOP.

Previous studies have shown that KDB surgery causes transient hyphema in 12.5–34.9% of patients and IOP spikes in 1.0–14.0% [21,23,25,27,28]. Hyphema occurred in 14.2% of the patients in our study but cleared spontaneously without intervention. IOP spikes occurred in 12.8% of eyes in our study, which is in line with rates in previous studies. IOP spikes occurred more often in eyes with ExG than in those with POAG (23.7% vs. 9.1%). Patients with ExG tend to have higher rates of elevated IOP than those without ExG after cataract surgery [29]. This tendency might have contributed to the significant difference in IOP spikes between the two groups. The number of additional glaucoma surgeries in our study was higher in ExG eyes (10.5%) than in POAG eyes (1.8%). ExG tends to be clinically more severe than POAG [30,31]. In our study, the preoperative IOP value was significantly higher in the ExG group than in the POAG group. Therefore, in the present study, eyes with ExG might have required reoperations of glaucoma more frequently than eyes with POAG.

Lower preoperative IOP was associated with surgical failure for criterion A in the present study. This result is consistent with the previous study [32]. The previous review article showed that ab interno trabeculotomy surgeries provided relatively good results when the target IOP is approximately 15–19 mmHg [33]. Therefore, it is difficult for eyes with lower preoperative IOP to achieve a ≥ 20% IOP reduction postoperatively.

Our study has some limitations, which stem mainly from its retrospective nature. First, we could not standardize preoperative characteristics, particularly in terms of the severity of glaucoma and cataract. Severe glaucoma is usually refractory to surgical treatment; therefore, differences in severity may have affected the surgical outcomes. Furthermore, postoperative inflammation may have been more severe because cataracts may have been more advanced. Second, some clinical data were lacking. Postoperative inflammation in the anterior chambers and postoperative peripheral anterior synechia may have affected the surgical outcomes because of increased resistance to aqueous outflow [34,35]. We acknowledge that gonioscopic examinations and measurement of flare values using a flare cell meter should have been performed. A prospective study would be required in the future to address these limitations.

## 5. Conclusions

A KDB procedure combined with cataract surgery achieved a significant reduction in IOP and the number of glaucoma medications required during long-term follow-up in Japanese patients with open-angle glaucoma. Eyes with ExG may have a higher complication rate than those with POAG after a KDB procedure and require more careful postoperative management.

## Figures and Tables

**Table 1:** Preoperative patient characteristics.

	Total	POAG	ExG	*p*-Value
Eyes, n	148	110	38	
Age (years)	76.9 ± 7.2	76.1 ± 7.4	79.0 ± 6.2	0.08
Sex, n (%)				0.09
Male	71 (48)	48 (44)	23 (61)	
Female	77 (52)	62 (56)	15 (39)	
Glaucoma type, n (%)				NA
POAG	110 (74)	NA	NA	
ExG	38 (26)	NA	NA	
Severity, n (%)				0.24
Mild	36 (24)	29 (26)	7 (18)	
Moderate	28 (19)	23 (21)	5 (13)	
Severe	84 (57)	58 (53)	26 (69)	
BCVA (logMAR)	0.53 ± 0.46	0.56 ± 051	0.44 ± 0.27	0.42
Spherical equivalent (diopters)	−2.6 ± 5.3	−3.0 ± 5.8	−1.6 ± 3.3	0.57
Axial length (mm)	24.0 ± 2.1	24.1 ± 2.2	23.8 ± 1.7	0.54
Visual field MD (dB)	−14.9 ± 9.3	−14.3 ± 9.3	−16.8 ± 9.4	0.17

Data are shown as the mean ± standard deviation. BCVA, best corrected visual acuity; ExG, exfoliation glaucoma; logMAR, logarithm of minimum angle of resolution; n, number; MD, mean defect; NA, not applicable; POAG, primary open-angle glaucoma.

**Table 2:** Intraocular pressure and the number of glaucoma medications before surgery and at the postoperative follow-up visits.

	Total	POAG	ExG	*p*-Value
Preoperative				
IOP (mmHg)	19.5 ± 6.9	18.5 ± 6.5	22.4 ± 7.4	<0.01
Medications, n	2.4 ± 1.4	2.4 ± 1.4	2.7 ± 1.5	>0.99
Eyes, n	148	110	38	
1 month				
IOP (mmHg)	14.0 ± 4.1	13.7 ± 3.6	14.9 ± 5.2	>0.99
Medications, n	1.2 ± 1.6	1.0 ± 1.5	1.8 ± 1.7	0.052
Eyes, n	148	110	38	
6 months				
IOP (mmHg)	13.1 ± 3.3	13.0 ± 3.3	13.6 ± 3.3	>0.99
Medications, n	1.4 ± 1.6	1.2 ± 1.6	2.0 ± 1.5	0.039
Eyes, n	138	102	36	
12 months				
IOP (mmHg)	12.9 ± 3.0	12.6 ± 2.9	14.0 ± 3.3	0.39
Medications, n	1.6 ± 1.5	1.4 ± 1.5	2.0 ± 1.5	0.50
Eyes, n	122	94	28	
24 months				
IOP (mmHg)	12.3 ± 2.7	12.1 ± 2.8	13.2 ± 2.3	0.71
Medications, n	1.7 ± 1.7	1.6 ± 1.7	2.0 ± 1.4	>0.99
Eyes, n	109	86	23	
36 months				
IOP (mmHg)	11.9 ± 2.7	11.4 ± 2.6	13.4 ± 2.3	0.014
Medications, n	1.6 ± 1.4	1.4 ± 1.4	2.1 ± 1.4	0.28
Eyes, n	93	70	23	
IOP reduction in baseline at 36 months (%)	28.0 ± 20.8	28.7 ± 20.1	26.0 ± 23.3	0.47
IOP reduction ≥20% in baseline at final visit, n (%)	105 (71)	81 (74)	24 (63)	0.22
Medication-free at 12 months, n (%)	47 (39)	41 (44)	6 (21)	0.046
Medication-free at 24 months, n (%)	39 (36)	35 (41)	4 (17)	0.051
Medication-free at 36 months, n (%)	32 (34)	28 (40)	4 (17)	0.075
Using ≥1 fewer medications from baseline at final visit, n (%)	83 (56)	67 (61)	16 (42)	0.058

Data are shown as the mean ± standard deviation. ExG, exfoliation glaucoma; IOP, intraocular pressure; n, number; POAG, primary open-angle glaucoma.

**Table 3:** Postoperative complications.

n (%)	Total	POAG	ExG	*p*-Value
Hyphema	21 (14.2)	15 (13.6)	6 (15.8)	0.79
IOP spikes	19 (12.8)	10 (9.1)	9 (23.7)	0.045
Additional glaucoma surgery	6 (4.1)	2 (1.8)	4 (10.5)	0.038

ExG, exfoliation glaucoma; IOP, intraocular pressure; KDB, Kahook Dual Blade; n, number; POAG, primary open-angle glaucoma.

**Table 4:** Multivariable analysis to identify prognostic risk factors for failure using Cox proportional hazards regression models.

	Criterion			
	A		B	
	RR (95% CI)	*p*-Value	RR (95% CI)	*p*-Value
Sex (Male/Female)	0.93 (0.52–1.65)	0.81	0.86 (0.53–1.39)	0.55
Age per year	0.97 (0.93–1.01)	0.13	0.97 (0.94–1.00)	0.08
Axial length per mm	1.04 (0.89–1.19)	0.63	1.04 (0.92–1.16)	0.54
Visual field MD per dB	0.99 (0.96–1.03)	0.7	0.98 (0.96–1.01)	0.23
Glaucoma type (POAG/ExG)	0.74 (0.39–1.48)	0.38	1.32 (0.76–2.37)	0.33
Preoperative IOP per mmHg	0.89 (0.83–0.95)	<0.01	1.02 (0.98–1.06)	0.31
Preoperative glaucoma medication per each	1.04 (0.85–1.27)	0.72	1.05 (0.89–1.24)	0.57
IOP spikes	1.41 (0.64–2.87)	0.38	1.46 (0.73–2.71)	0.27

ExG, exfoliation glaucoma; IOP, intraocular pressure; MD, mean defect; POAG, primary open-angle glaucoma; RR, relative risk.

## Data Availability

Data is fully available upon reasonable request to corresponding author.
